# Did Pterosaurs Feed by Skimming? Physical Modelling and Anatomical Evaluation of an Unusual Feeding Method

**DOI:** 10.1371/journal.pbio.0050204

**Published:** 2007-07-24

**Authors:** Stuart Humphries, Richard H. C Bonser, Mark P Witton, David M Martill

**Affiliations:** 1 School of Biological Sciences, University of Reading, Reading, United Kingdom; 2 School of Construction Management and Engineering, University of Reading, Reading, United Kingdom; 3 School of Earth and Environmental Sciences, University of Portsmouth, Portsmouth, United Kingdom; University of Lund, Sweden

## Abstract

Similarities between the anatomies of living organisms are often used to draw conclusions regarding the ecology and behaviour of extinct animals. Several pterosaur taxa are postulated to have been skim-feeders based largely on supposed convergences of their jaw anatomy with that of the modern skimming bird, Rynchops spp. Using physical and mathematical models of *Rynchops* bills and pterosaur jaws, we show that skimming is considerably more energetically costly than previously thought for *Rynchops* and that pterosaurs weighing more than one kilogram would not have been able to skim at all. Furthermore, anatomical comparisons between the highly specialised skull of *Rynchops* and those of postulated skimming pterosaurs suggest that even smaller forms were poorly adapted for skim-feeding. Our results refute the hypothesis that some pterosaurs commonly used skimming as a foraging method and illustrate the pitfalls involved in extrapolating from limited morphological convergence.

## Author Summary

Just because a component of an extinct animal resembles that of a living one does not necessarily imply that both were used for the same task. The lifestyles of pterosaurs, long-extinct flying reptiles that soared ancient skies above the dinosaurs, have long been the subject of debate among palaeontologists. Similarities between the skulls of living birds (black skimmers) that feed by skimming the water surface with their lower bill to catch small fish, and those of some pterosaurs have been used to argue that these ancient reptiles also fed in this way. We have addressed this question by measuring the drag experienced by model bird bills and pterosaur jaws and estimating how the energetic cost of feeding in this way would affect their ability to fly. Interestingly, we found that the costs of flight while feeding are considerably higher for black skimmers than previously thought, and that feeding in this way would be excessively costly for the majority of pterosaurs. We also examined pterosaur skulls for specialised skimming adaptations like those seen in modern skimmers, but found that pterosaurs have few suitable adaptations for this lifestyle. Our results counter the idea that some pterosaurs commonly used skimming as a foraging method and illustrate the pitfalls involved in extrapolating from living to extinct forms using only their morphology.

## Abstract 3

Physical and mathematical models of fossil pterosaurs and a living bird that feeds by skimming refute the hypothesis that pterosaurs would have been able to forage this way.

## Introduction

Much of our understanding of extinct organisms comes from anatomical comparisons with extant species. Similarities in morphology are often used to reach conclusions about the ecology and behaviour of long-vanished animals [1–7] as well as their susceptibility to extinction events [8]. This approach is certainly a valid one in many cases, but its very appeal can sometimes lead to its misuse. Here we show that assumptions about morphological convergence have led to a widespread, but erroneous, belief regarding the feeding behaviour of pterosaurs (extinct flying reptiles).

Despite considerable interest in the biomechanics of flight among pterosaurs, the feeding methods of these animals are still poorly understood. This situation is in stark contrast to other Mesozoic reptiles in which the feeding apparatuses have been subject to numerous investigations in functional morphology and biomechanics [9–11]. Based on postulated convergence in mandible morphology, several pterosaurs are suggested to have fed by skimming; the activity of flying low over water with the tip of the lower mandible immersed and seizing prey items on contact [2,6,7,12–16]. This foraging technique is seen in the extant “skimmers” (Aves: Rynchopidae), in which all three *Rynchops* species forage almost exclusively by skim-feeding (Figure 1) [17]. Skimming-like behaviour has been documented occasionally for a few terns and gulls [18,19], but habitual foraging using this method has been observed only in *Rynchops*. The skimming behaviour of *Rynchops* is well documented and is most effective in areas of calm, shallow water, and where aggregations of fish or small crustaceans are abundant near the water surface [17,20–24]. Although skimmers do occasionally capture fish using alternative methods [25], their ability to capture prey without skimming is limited [22].

**Figure 1:**
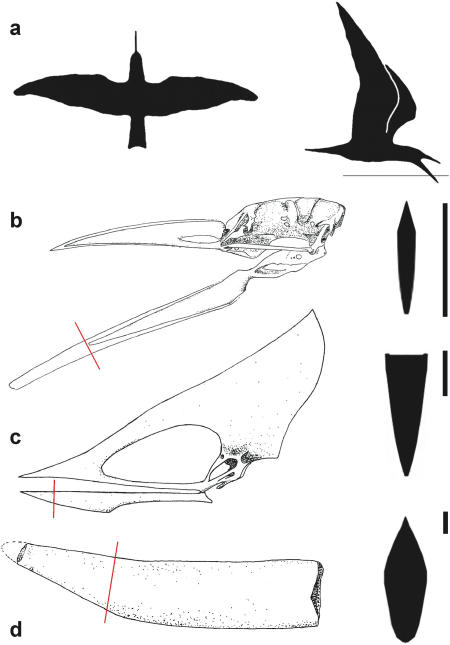
Comparative Dimensions of *Rynchops* and Pterosaur Lower Jaws (A) Dorsal (left) and lateral (right) views of *Rynchops niger cinerascens* in foraging flight. Flight altitude was estimated from bill length, bill penetration and bill angle (45 °). (B) Skull of *Rynchops* showing cross-sectional shape of lower jaw. (C) Skull of Tupuxuara sp. (IMCF 1052) and cross-section of lower jaw. (D) Jaw fragment of Thalassodromeus sethi (DGM 1476-M) and cross-section of lower jaw. Lines of sections indicated by red lines. Jaw section scale bars equal to 10 mm.

Many authors have discussed skim-feeding in pterosaurs, but the hypothesis remains controversial. The basal pterosaur *Rhamphorhynchus* was the first postulated pterosaur skimmer [12]. Mateer [4] considered that *Pteranodon* used a skimming-like technique to scoop plankton and other small creatures from the water surface during flight. A headcrest-anchored muscle was hypothesised to resist depressive forces acting on the jaw during this process [4]. Wellnhofer [7,16] echoed these suggestions in also suggesting that *Pteranodon* and *Rhamphorhynchus* were skimmers.

Nesov [15] inferred that pteranodontian and ornithocheirid pterosaurs were skimmers when discussing the skimming capability of the azhdarchid *Azhdarcho*. Nesov [15] further suggested that the long azhdarchid neck would have been useful in skimming by allowing the animal to reach food at shallow depths as well as at the water surface. Martill [14] also suggested a long neck would be useful in skimming by permitting flapping of the wings while feeding without the wingtips touching the water surface. Further support for azhdarchid skimming was given by Kellner and Langston [13] and by Prieto [6] when considering the giant pterosaur *Quetzalcoatlus*. Kellner and Langston [13] stated that the maximum jaw gape of *Quetzalcoatlus* was “in keeping with the notion of fishing on the wing, somewhat in the fashion of the existing skimmer, *Rynchops*” and that skimming is generally “more plausible than Lawson's (1975) carrion feeding hypothesis.” *Thalassodromeus* has recently been described as a likely pterosaurian skimmer after its apparent mandibular similarities with *Rynchops* [2]. These authors further suggest that the jaw morphology of *Thalassodromeus* almost entirely precludes all other feeding methods.

However, the skim-feeding hypothesis also has detractors. Bennett [26] suggested that *Pteranodon* lacks hooked jaws to capture or grasp fish whilst in flight. Ősi et al. [27] hypothesised that the long, rigid necks and slender mandibular rami of azhdarchids would limit their skimming capability. Chatterjee and Templin [1] speculated that large pterosaurs lacked the necessary flight power and manoeuvrability to skim-feed. These authors also highlighted distinctions between the blunted mandibular tips of skimmers and the pointed jaw tips of postulated pterosaur skimmers, suggesting that blunt tips may deflect water to either side of the jaw during skimming and reduce energy costs. Although advocating skimming in *Thalassodromeus*, Kellner and Campos [2] highlight that *Rhamphorhynchus* lacks many adaptations expected in skimmers and suggest it was a comparatively poor skim-feeder.

By using data from physical experiments with life-sized models of mandibles from suggested skimming pterosaur taxa and modern skimmers, we tested the idea that pterosaurs may have skim-fed. The results were then evaluated with both hydrodynamic and aerodynamic models. We used a bill cast of *Rynchops niger cinerascens* (a subspecies of R. niger), a model of a jaw fragment (Museu de Ciências da Terra of the Departmento Nacional de Produção Mineral, Rio de Janeiro, Brazil, accession number DGM 1476-M) attributed to the azhdarchoid pterosaur Thalassodromeus sethi [28], and for comparison, a pterosaur not thought to skim-feed, Tupuxuara sp. (Iwaki Coal and Fossil Museum, Iwaki, Japan, accession number IMCF 1052). Because of the assertion that the tip of the mandible of *Thalassodromeus* was laterally compressed [2], we also used hydrodynamic principles to model the drag resulting from a generic compressed bill with the characteristics of a reversed aerofoil. This modelling approach allowed us to assess the skimming potential of two other suggested [4,6] skim-feeding pterosaurs—*Pteranodon* and *Quetzalcoatlus*—as well as the two known specimens of *Thalassodromeus* [2,28].

## Results

### Drag Estimation

Over the range of velocities used (1.8–6.8 m s^−1^), the *Rynchops* bill cast experienced an approximately 14-fold increase in drag from 0.05–0.72 N (Figure 2A). The *Tupuxuara* model, immersed to the same depth (0.04 m), experienced drag that was nearly an order of magnitude higher at 0.30–5.18 N, with the data for *Thalassodromeus* of the same order (Figure 2B). The *Rynchops* data from the flume trials were modelled using an ordinary least-squares regression (OLS) of log-transformed drag on log-transformed velocity (0.286*V* ^1.52^, *n* = 17 flume runs, adjusted *r* ^2^ = 0.76, *p* < 0.001; Figure 2). To provide a conservative estimate of drag, the equation for the lower 75% confidence interval of the OLS was used to describe drag of the *Rynchops* bill (0.019*V* ^1.26^). Comparison of the thin plate approximation (Equations 1–6) of a *Rynchops* mandible with the lower 75% CI line of an OLS regression of drag on velocity for the physical model (Figure 2A) indicates that our hydrodynamic model captures the behaviour of the physical model appropriately and thus supports our use of this technique for the mandible of large pterosaurs. Drag measurements for the model *Rynchops* bill and both Tupuxuara sp. and the *Thalassodromeus* jaw fragment accord well with estimates derived from fluid dynamic principles to give conservative estimates of drag (Figure 2B).

**Figure 2:**
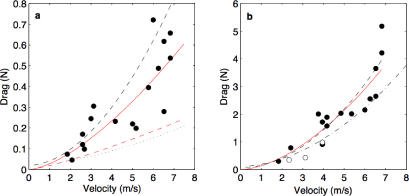
Drag Measurements and Model Predictions (A) Drag measurements for a resin cast of a *Rynchops niger cinerascens* mandible. Solid red line is an OLS of log-transformed data, broken red line is the lower 75% confidence interval for the OLS. Broken black lines are drag estimates from fluid-dynamic models (dashed: aerofoil; dotted: thin plate approximation). (B) Drag measurements for *Tupuxuara sp.* mandible model (filled circles), and that of a mandible attributed to *Thalassodromeus* (open circles). The solid line is a OLS for log-transformed *Tupuxuara* data, dashed line the drag estimate from fluid-dynamic models (aerofoil).

Estimated flight costs including hydrodynamic drag were compared against an estimate of available metabolic power from Marden [29], using the hydrodynamic model for the pterosaurs to adjust immersion depth to 19%. Estimated minimum cost of flight (minimum power speed *V*_mp_) and minimum cost of flight per metre travelled (maximum range speed *V*_mr_) were 5.9 m s^−1^ and 11.0 m s^−1^ respectively for *Rynchops* (Figure 3A and 3B). Flight costs were considerably higher with *V*_mp_ at 3.9 m s^−1^ and 4.3 m s^−1^, and *V*_mr_ at 6.2 m s^−1^ and 6.9 m s^−1^ for *Tupuxuara* (Figure 3C and 3D) and *Thalassodromeus* (fragment; Figure 3E and 3F), respectively. The estimated foraging flight costs for powered flight (Figure 3) show both the reduced costs at intermediate speeds and the rapidly increasing costs at very low speed (resulting in higher stall speeds) associated with wing-in-ground effect flight [30]. When considered in relation to an estimate of maximal metabolic power available for powered flight based on body mass of flying vertebrates [29], *Rynchops,* despite high hydrodynamic drag costs (Table 1), is still capable of foraging for long periods with its bill submerged to a depth of 0.04 m. Neither of the pterosaurs appears able to meet the energetic costs of skimming. The fact that the observed flight speed of 10 m s^−1^ for *Rynchops* [19,31] is closer to the theoretical *V*_mr_ suggests that the bird is attempting to reduce the cost of transport, and this finding supports the idea that foraging flight in this species maximises the distance travelled for a given energy input [31,32].

**Figure 3:**
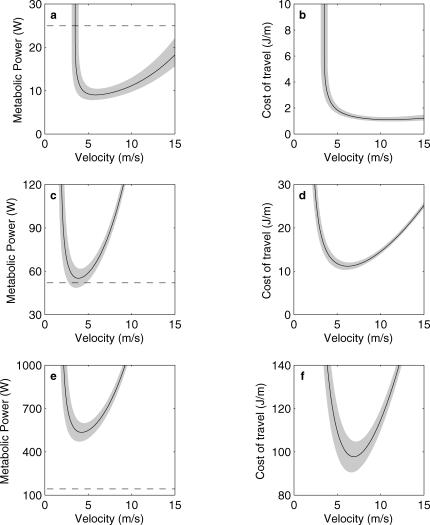
Power Curves (A, B) *R. n. cinerascens,* (C, D) Tupuxuara sp., and (E,F) *Thalassodromeus* in flapping flight. Plots are of horizontal flight speed versus metabolic flight costs in ground effect with the mandible immersed to the depth indicated in Table 1 (A, C, and E), and metabolic costs per metre travelled (B, D, and F). Shaded areas bound the limits of the high- and low-power estimates from the sensitivity analysis. The horizontal line on A, C, and E indicates estimated available metabolic power (47.126*M* ^0.605^) [29])

**Table 1:**
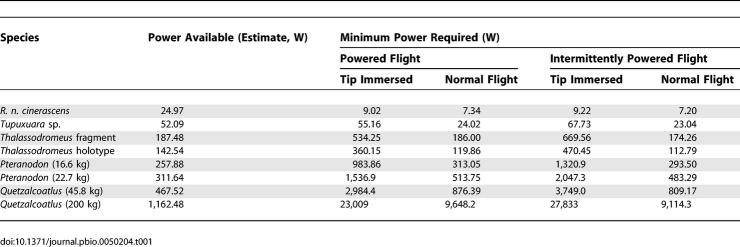
Estimated Flight Costs for Both Powered and Intermittently Powered Flight with and without the Tip of the Mandible Immersed

The added costs of flight due to hydrodynamic drag range from 20% of total costs in *Rynchops*, up to 68% in the low-mass estimate for *Pteranodon* (Table 1, Figure 4). Among the pterosaurs, even *Tupuxuara,* which seems potentially able to fly with the tip of its mandible immersed, uses half of the energy needed for flight on hydrodynamic drag. It is clear that in all species, hydrodynamic drag constitutes a major component of total flight costs. It is also clear that with hydrodynamic drag costs incorporated, the benefits of intermittently powered flight are negated for most species (Table 1).

**Figure 4:**
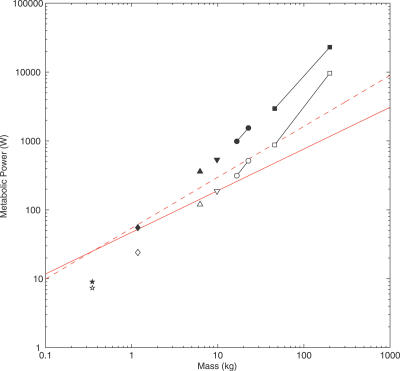
Estimated Metabolic Costs Estimated metabolic costs of steady flapping flight in ground effect (*V*_mp_) with (filled symbols) and without (open symbols) the tip of the mandible immersed in water. Stars, *R. n. cinerascens;* diamonds, *Tupuxuara sp.;* up-triangles, *Thalassodromeus* fragment; down-triangles, *Thalassodromeus* holotype; circles, *Pteranodon;* squares, *Quetzalcoatlus*. Solid red line indicates estimated maximum available metabolic power (47.126*M* ^0.605^) [29], broken red line the theoretically maximum available metabolic power (54.144*M* ^0.739^) [29].

## Discussion

### Rynchops

Our results allow us to re-evaluate the foraging ecology of *Rynchops.* The hydrodynamic drag on the bill is considerably greater than that previously estimated by Withers and Timko [19]. In the context of our revised estimates of the costs involved in foraging flight, and contrary to the only other previous study [19], we found that hydrodynamic drag from the bill tip has significant implications for the energy budget of *Rynchops*. At the typical recorded foraging speed of R. niger (10 m s^−1^ [19,31]), the drag extrapolated from our measurements for the model *R. n. cinerascens* (0.35 N) is at least three orders of magnitude greater than that previously estimated [19] (1 × 10^−4^ N). We attribute this disparity to inadequate consideration of dynamic scaling methods in [19] as accurate estimation of drag at the water surface requires test velocities close to those in real life to satisfy scaling of both the Reynolds number *(Re)* and Froude number *(Fr)* [33].

On the basis of the calculations provided by Withers and Timko [19], it has generally been assumed that the hydrodynamic costs of skimming are trivial compared to the aerodynamic costs of flight (e.g., [17,31–33]). Similar to our work, Withers and Timko [19] suggested that wave drag on the lower mandible could safely be ignored (see Materials and Methods), but they did not consider spray drag at all. Nonetheless, although we demonstrate that spray drag is less important than pressure drag, it is still an important component of the hydrodynamic drag costs. For the thin-plate approximation of the *Rynchops* bill, we find that as a percentage of total drag costs, the drag components contribute 79.1% (pressure), 13.5% (ventilation), and 7.4% (spray) at *V*_mp_. However, whereas Withers and Timko [19] concluded that hydrodynamic costs for skimmers are less than 0.1% of the aerodynamic costs of flight, we argue that correct measurement of drag (matching *Re* and *Fr*) suggests that hydrodynamic costs are two orders of magnitude higher than that estimate (20% of aerodynamic costs). To this end, we suggest that the high energetic requirements of this foraging technique, despite considerable morphological specialisation, may explain its rarity in extant birds.

### Pterosaur Biomechanics

Hydrodynamic drag estimates for pterosaur mandibles are considerably higher than those of *Rynchops*, and in the context of aerodynamic estimates rule out skim-feeding for those animals heavier than 2 kg. Based on the flume results, our modelling appears to describe well the hydrodynamics of an immersed *Rynchops* bill tip and lends support to our use of the model for pterosaur jaws. In accordance with current thinking, powered flight seems an unlikely proposition for *Quetzalcoatlus,* even without the added drag from skim-feeding. Second, even with the conservative hydrodynamic drag estimates used, our calculations suggest that neither *Thalassodromeus, Pteranodon,* or *Quetzalcoatlus* would have been able to use skimming as a feeding method in continuous flight or with intermittent flapping, although *Tupuxuara* may have been able to meet skim-feeding energy requirements based on its proximity to its theoretical available metabolic power (Figure 4, broken red line). However, the estimated required power output is still around 10 W more than the other estimate of available power, based on empirical data from Marden [29]. This is a somewhat surprising finding, because Tupuxuara sp. was not a species we expected to be able to skim-feed, but the result most likely reflects our conservative modelling of drag. However, it does also present a possible explanation for evolution of the skimming habit. If, as some authors predict (e.g., [1,34]) pterosaurs had lower wing loading than extant birds, they were probably able to cope with some additional flight costs. If the ancestors of modern skimmers were either smaller or had proportionally larger wings, this may have given them the ability to tolerate the increased costs of skimming with a “normally” shaped bill and to take the step from dipping for prey to some kind of skimming behaviour. It also leaves the theoretical possibility that smaller (<2-m wingspan) pterosaurs such as *Rhamphorhynchus* may have been able to skim-feed occasionally without particularly specialised jaw morphology due to their lower flight costs (through lower wing loading [34]) than extant birds.

Our analyses assume air density at current levels, but this value may not have been constant over geological time [35,36]. Because we use gross estimates of available power, we are unable to estimate directly the implications for lift and drag of air with differing densities. However, if we assume a direct relationship between air density and lift, then over the relevant timescale for pterosaurs, the density minimum of the Triassic (87% of present [36]) and the density maximum of the early Tertiary (121% of present [36]) might be expected to increase or decrease the power requirements for flight by 13% and 21% respectively. Neither of these values is likely to change the overall conclusions presented here.

### Pterosaur Osteology

To date, analysis of potential skim-feeding adaptations in pterosaurs have been limited, typically focusing on single cranial or cervical features rather than considering the entire skull and neck. This suggests a misunderstanding of just how specialised skim-feeding is: modern skimmers require numerous adaptations across their cranial anatomy to facilitate efficient skimming and to resist forces generated during feeding [24,37]. Some 30 adaptations are seen in the skull and neck of *Rynchops* that cope with both hydrodynamic forces acting upon the skull whilst skimming and with the impact of capturing prey, as well as maintaining bill position within the water column, detecting prey, and regenerating abraded bills [24,37]. The most obvious of these adaptations include extreme lateral compression and pronounced horny extension of the mandibular symphysis, large jaw muscles, a reinforced quadrate-articular joint resistant to lateral disarticulation, developed medial bracing of the mandible, short mandibular rami, elongate mandibular symphysis, and a segregated, highly flexible neck [24,37]. Such adaptations are not seen in other birds and are thought to represent specialisations to the highly derived lifestyle of skimmers [24].

We acknowledge that pterosaurs may have possessed skimming adaptations distinct from those of *Rynchops,* but the structures of pterosaur and bird skulls are not so different that some functional convergence should be expected if, indeed, pterosaurs did skim. However, few of the adaptations expected are seen in these postulated skimming taxa. For instance, pterosaur skulls are often elongated, but not one pterosaur yet found shows the degree of lateral compression seen in the mandible of *Rynchops*. Relatively pronounced lateral compression of the mandibular symphysis is seen in *Pteranodon, Rhamphorhynchus,* and *Thalassodromeus,* with tapered dorsal and ventral symphyseal surfaces also seen in the latter taxa [2,26,38], but none exhibit the extreme condition of this feature seen in *Rynchops* (Figure 1).

The rarity of soft tissue preservation limits understanding of pterosaur jaw soft tissues, and it remains possible that some postulated pterosaur skim-feeders could have possessed horny mandibular sheaths similar to those of *Rynchops* to extend the jaw and pronounce lateral compression. This structure appears vitally important for skimming *Rynchops,* because it extends the feeding depth and provides an expendable jaw tip that can be replaced if damaged accidentally on submerged obstacles or grounded on substrata [24]. A review of *Rynchops* literature suggests that such accidents may be relatively common [17]. The rhampotheca of *Rynchops* is highly vascular and grows continuously, presumably to regenerate the abraded or broken bill tips [24]. Such an adaptation, therefore, seems crucial to skim-feeding. However, no known pterosaurs show a mandibular sheath comparable to that of *Rynchops*. *Rhamphorhynchus* does show a soft-tissue mandibular extension, but it is considerably shorter than that of skimmers and shows dorsal curvature [38] instead of ventral as seen in skimmers.

Several pterosaurs are skimmer-like in possessing short mandibular rami (e.g., *Pteranodon* and *Quetzalcoatlus,* though not *Thalassodromeus*), but their rami differ from *Rynchops* in being relatively slender. This suggests that pterosaurs had comparatively little mandibular anchorage for their jaw adductor musculature, which is known to be large in *Rynchops*. This increased muscle mass in *Rynchops* is critical for stabilising the mandible and counteracting depressive forces acting on the mandible whilst skim-feeding [24]. Skimming pterosaurs would almost certainly require similarly developed musculature to forage successfully.

*Rynchops* possesses a reinforced jaw joint supported by bony bracing at the quadrate and the basitemporal plate [24,37]. Several other bird groups demonstrate a similar condition, but none show the development of this bracing to the level seen in *Rynchops* [37]. Reinforcement of the jaw is thought to be necessary to withstand the impact phase of skim-feeding [24], and no pterosaur yet known shows jaw bracing of this kind. The jaw articulator facets of *Rhamphorhynchus* and *Tupuxuara* are deep (DM Unwin, personal communication) and a small laterally bracing “peg” is known in *Quetzalcoatlus* [13], but despite this, pterosaur jaw joints are considerably less robust than those of *Rynchops*.

The neck of *Rynchops* is also subjected to large stresses during skim-feeding [24], including caudoventral forces on the skull and neck that are countered by powerful neck muscles anchored to a flexible, robust cervical series. The cervical vertebrae are segmented into articulatory groups and highly sculpted to situate enlarged neck musculature [24], with the anterior vertebrae particularly robust. Such adaptations are needed when an impact occurs during skimming, as these force the head and anterior cervical segments to rotate caudoventrally. Strong, flexible necks appear critical to absorbing impact forces while skim-feeding.

Amongst pterosaurs, the cervicals of *Rynchops* are most distinct from those of azhdarchids. These pterosaurs bear slim, elongate cervicals with broad, interlocking zygopophyses and reduced or absent neural spines and transverse processes [39]. Many authors have noted the inflexibility of the azhdarchid cervical series (e.g., [27,40,41]), and this would appear to be a severe handicap to a skim-feeder. So great are these distinctions in cervical vertebrae between azhdarchids and *Rynchops* that we echo the suggestion of Ősi et al. [27] that neck osteology alone casts serious doubt on the skimming potential of azhdarchids. The cervical vertebrae of *Rhamphorhynchus, Pteranodon,* and tupuxuarids compare more favourably with those of skimmers in being relatively robust and bearing developed processes. The biomechanics of pterosaur necks are still largely uninvestigated, but we note that neither *Rhamphorhynchus* nor *Pteranodon* show the distinct segmentation and size increase of the anterior cervical series seen in *Rynchops* (Figures 27 and 28 of [24]). The cervical series of tupuxuarids are among the most robust and flexible of all pterosaurs (DM Unwin, personal communication), but we also note that many feeding strategies require flexible necks, not just skimming. Hence, these features of tupuxuarid anatomy do not necessarily advocate skim-feeding habits in these forms.

Almost without exception, pterosaur anatomy appears poorly adapted for skim-feeding. Comparisons between pterosaurs and *Rynchops* reveal little of the convergence expected between animals postulated to have such similar, specialist lifestyles, with the relatively wide mandibular symphyses, apparent absences of elongate, abradable mandibular sheaths (at least observable in *Rhamphorhynchus*), and lack of a suitably reinforced jaw joint particularly pertinent arguments against pterosaur skimming. We note that *Dsungaripterus,* a pterosaur specialized for crushing hard-shelled foods [16], shares an upturned, dorsally tapered mandibular symphysis with many of the postulated skimming pterosaurs discussed here. Upturned jaws may provide increased grip on food through the larger occlusion surface with the bolus along the lower jaw. Straight jaws, by contrast, can only hold an object at a single point on each jaw. Dorsally tapered symphyses may also allow for greater application of pressure on food items and do not necessarily indicate skimming habits.

### Conclusions

The idea that pterosaurs were skimmers appears to have originated through limited morphological comparisons with modern forms (e.g., [2]) and inadequately investigated functional inferences (e.g., [15]). Both modelling the energy requirements of skimming pterosaurs and analysing their osteology casts serious doubt on the ability of pterosaurs to habitually skim-feed. Although our physical modelling suggests that small pterosaurs may have been energetically capable of skimming, there is no anatomical evidence to assume that *Rhamphorhynchus* or any other small pterosaurs were skimmers. From this, we stress the difficulty of using limited morphological convergence to interpret the ecology of extinct forms: comprehensive analysis of this nature can be insightful but should ideally be supported with additional biomechanical data.

## Materials and Methods

### Drag measurements using physical models.

Model bills were cast in epoxy resin (*R. n. cinerascens*, BMNH 1914.12.2.9), or modelled with dense, varnished, polyethylene foam (*Tupuxuara sp.*, [IMCF 1052] and *Thalassodromeus* fragment [DGM 1476-M]) from specimens and data [28]. Model dimensions are given in Table 2. These physical models were instrumented with strain gauges connected to an amplifier, with the output signals recorded on a computer. Models were suspended from a trolley towed above a water flume (0.5 m width × 12.0 m length, 0.25-m water depth, 8.0-m trolley travel) at different speeds selected from the known range for *Rynchops* and determined by speed settings for the towing motor. Velocity data were extracted from video footage of the trials (Sony DCR HC1000, 25 frames per s) and bending moments present at the strain gauges were used to calculate the drag force acting on the models.

**Table 2:**
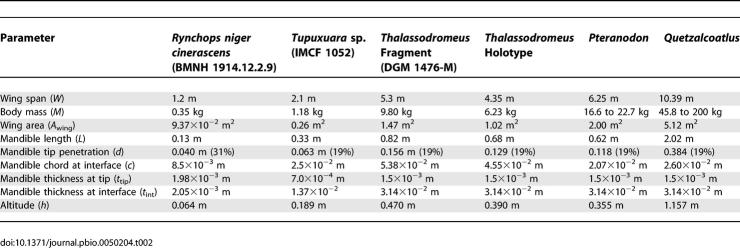
Key morphological parameters

### Drag modelling.

In physical terms, the laterally compressed mandible of *Rynchops* acts as a surface-piercing strut, and as such, the drag that it experiences is potentially comprised of five additive components: friction drag, pressure drag, wave drag, spray drag, and drag due to ventilation [42]. Of these five components, the latter three are specific to surface-piercing struts and are the result of energy losses from work performed in displacing water vertically against the force of gravity [42].

Drag coefficients representing pressure and friction drag vary with Reynolds number *(Re)*. The distal section of the mandible of *Rynchops* is similar to a reversed streamlined airfoil (e.g., NACA 0012), for which data collected at *Re* ≥ 10^6^_,_ suggests coefficients of drag twice that of a typical streamlined airfoil oriented with its blunt leading edge facing the direction of movement [33,42]. Such sections are apparently adapted to reduce drag components associated with the air–water interface [33,43]. We have no reason to believe that the coefficient of drag for such a reversed airfoil will vary with *Re* in the same way as a conventionally oriented airfoil will, especially at *Re* < 10^6^, a range that has received comparatively less attention from fluid dynamicists. Although we know almost nothing about the behaviour of reversed airfoils, Hoerner [42] suggests that the coefficient of drag for sharp-nosed foil sections (i.e., thickness ratios less that 15%) at *Re* < 10^6^ may behave in a manner similar to that of a thin plate in laminar flow. Composite simulation and experimental data from work on turbine blades supports this theory by suggesting the drag coefficient of a reversed NACA 0012 airfoil is approximately constant over the range 10^4^ ≤ *Re* ≤ 10^7^ [44]. We thus describe the *Re* dependence of *Cd*_pro_ (coefficient of profile drag, including pressure and friction components) of the tip of a *Rynchops* mandible by using the theoretical drag coefficient for a thin plate in laminar flow corrected for a drag coefficient based on frontal area

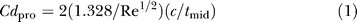
where 1.328/Re^1/2^ is the coefficient of skin friction drag for laminar flow *C*_fL_ [42]. The ratio *c*/*t*_mid_ is the inverse of the thickness ratio *t*_mid_/*c* where *t*_mid_ is the thickness of the plate at the midpoint between its tip and the water surface and *c* is the chord of the plate at the water surface. We calculate profile drag as

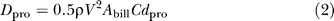
where ρ is the density of water, *V* the velocity of the mandible, and *A*_bill_ is the projected frontal area of the bill, from its tip to the water interface.

We now turn to the spray and wave drag components associated with the breaking of the water surface by the mandible. Both wave and spray drag depend on the Froude number *(Fr)*, which is a measure of the ratio of inertial and gravitational forces and is used to compare the wave-making resistance between bodies of various sizes and shapes. Froude number, based on the chord of the mandible, is given by

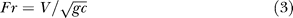
where, as before, *V* is the speed through the water, *g* is the acceleration due to gravity (9.81 m s^−2^), and *c* is the chord of the mandible at the water surface. Spray drag effects are unfortunately poorly described at 1 ≤ *Fr* < 3 [42]. However, we do know that the coefficient of spray drag (*Cd*_spray_, based on spray drag divided by mandible thickness-squared [42]) is relatively constant and of the order 0.24 for *Fr* ≥ 3 for semi-streamlined double arc sections with forebody thickness ratios (*t/x*) ≤ 0.4 (where *x* is forebody length, from leading edge to point of maximum thickness on a double arc or aerofoil section; Figure 25, section 10–13 of [42]. Both the mandible of *Rynchops* and the NACA 0012 airfoil fall into this category, and even at 1 m s^−1^ for the *Rynchops* bill, *Fr* = 3.5. For struts at *Fr* < 3, the velocities involved are small and we assume that spray drag is negligible. The spray drag is thus given by

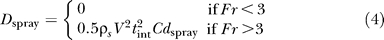



Wave drag represents the kinetic energy transferred from an object travelling at the water surface as water is accelerated upward [42]. Wave drag increases to a maximum at *Fr* ≈ 0.5 [42], but then decreases steadily to zero near *Fr* = 3. Thus for the mandible of *Rynchops*, wave drag is likely to be zero at velocities near 1 m s^−1^, and we consider its effects to be negligible in comparison to spray drag down to 0.5 m s^−1^ and therefore assume that it can be ignored.

The ventilation drag component is caused mainly by the presence of an area of negative pressure behind a moving object that draws air downwards and increases its effective chord [42]. Drag due to ventilation is very much dependent on the shape of the strut, being greater in those with flat trailing edges, but in general, the coefficient for drag due to ventilation can be given by

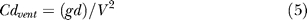
where *d* is the depth to which the tip of the mandible penetrates the water [42]. We therefore estimated the drag due to ventilation as

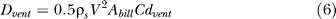



Finally, the three relevant drag components (*D*_pro_, *D*_spray_, *D*_vent_) were summed to give an estimate of the total drag (*D*_tot_) acting on the immersed tip of the mandible. We also used drag components calculated for a NACA 0012 aerofoil oriented with a blunt leading edge as a comparison to the flat plate approximation of a mandible tip, because its profile is more similar to that of the tips of the pterosaur mandibles. This implementation of the model differed from that given above, only in that *Cd*_pro_ was calculated (after [42]) as

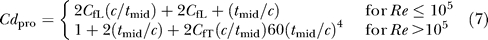
with *C*_fL_ from Equation (1), and *C*_fT_, the coefficient of skin friction drag for turbulent flow, well described by the “Schoenherr line”, an empirically derived equation to describe skin friction. The coefficient of skin friction drag for turbulent flow can be approximated within 2% by

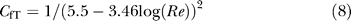
(Equation 26, section 2–5 of [42]). Throughout the modelling, the mandible was assumed to be angled at 45 °, and this inclination was accounted for by adjusting the drag function by a factor of 1.415 following the results of Withers and Timko [19].

Whereas *Rynchops* was treated as having the distal 31% of the mandible immersed, physical models for *Tupuxuara* and the *Thalassodromeus* fragment were, for logistic reasons, immersed to the same absolute depth (0.04 m) as the *Rynchops* cast. To account for the discrepancy, we modelled the pterosaur bills using the hydrodynamic model with an immersion depth of 19% of its length. This immersion was chosen as it represents the posterior region of the mandibular symphysis in the *Thalassodromeus* jaw and thus the point after which sudden lateral expansion of the mandible occurs.

Whereas data from the model jaw of *Tupuxuara sp.* could be used to estimate foraging flight costs, the jaw fragment attributed to *Thalassodromeus* [28] was considerably thicker than is suggested by Kellner and Campos [2]. In the absence of mandible dimensions other than length and chord, the dimensions of the *Thalassodromeus* fragment were used for both *Thalassodromeus* jaw models*,* as well as for those of *Pteranodon* and *Quetzalcoatlus* (Table 2).

### Pterosaur mass estimation.

To model flight costs, we estimated the body mass *(M)* and wing area (*A*_wing_) of pterosaur specimens in this study using a regression formula based on data on mass and wingspan *(W)* for the following 13 species of pterosaur for which wing area estimates were also available [34]: *Campylognathoides zitteli, Dorygnathus banthensis, Dsungaripterus weii, Eudimorphodon ranzi, Nyctosaurus gracilis, Pteranodon* sp*., Pterodactylus antiquus, P. elegans, P. kochi, P. micronyx, Rhamphorhynchus intermedius, R. muensteri,* and *Scaphognathus crassirostris*. In addition, we used data on wingspan and area for Tapejara wellnhoferi, Anhanguera piscator, and Quetzalcoatlus northropi from Chaterjee and Templin [1]. The dataset of Hazelhurst and Rayner [34] provides three estimates of wing area based on the postulated posterior attachment point of the wing membrane, whereas that of Chatterjee and Templin [1] provided only one, which fitted best when placed with Hazelhurst and Rayner's type III area (ankle attachment). OLS regressions of log(*M*) on log(*W*) and log(*A*_wing_) on log(*W*) all gave good fits (*M* = 0.229*W* ^2.215^, *n* = 13, *p* < 0.0001, adjusted *r^2^* = 0.978; *A*_wing1_ = 0.0668*W* ^1.854^, *n* = 13, *p* < 0.0001, adjusted *r^2^* = 0.992; *A*_wing2_ = 0.0886*W* ^1.778^, *n* = 10, *p* < 0.0001, adjusted *r^2^* = 0.990; *A*_wing3_ = 0.138*W* ^1.692^, *n* = 15, *p* < 0.0001, adjusted *r^2^* = 0.925). The estimated masses are given in Table 2.

Wingspan estimates for *Quetzalcoatlus* [1], the *Thalassodromeus* holotype [2], and Tupuxuara sp. (Witton and Martill, personal observation) were used to estimate other parameters for these species using the regressions generated from the Hazelhurst and Rayner data. The regression method produced estimates of mass for larger species that fell near the lowest published estimates, so to account for the variation in current mass estimates for large pterosaurs, we also used two published masses for *Pteranodon* and *Quetzalcoatlus* from the upper end of accepted estimates [45,46]. Wingspan for the *Thalassodromeus* specimen of Veldmeijer et al. [28] was estimated from the holotype [2] by scaling to the height of the mandibular symphysis.

### Modelling flight costs.

We estimated the metabolic power (*P*_met_) required for both flapping and flap-gliding flight using the methods of Pennycuick ([47] and summarised by [48]) and Norberg [49], respectively. There are three components to the power requirements for level flight: parasite power, induced power, and profile power. Following Ward et al. [50], the parasite power was estimated as

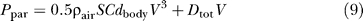
where ρ_air_ is the density of air (≈1.23 kg m^−3^), *S* is the projected frontal area of the animal's body (calculated as 8.13×10^−3^ *M* ^0.666^, after [47], but see also [51,52]), and *Cd*_body_ is an estimate of the coefficient of drag of the body. We use *Cd*_body_ = 0.25 in accordance with the revised estimates of Pennycuick et al. [53] for species with a prominent head and enlarged bill. The second term on the right hand side of Equation (9) represents the costs associated with ploughing the tip of the mandible through the water as a function of velocity.

The induced power was estimated as

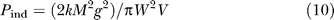
where *k* is an induced velocity scaling factor related to the degree of efficiency with which flapping wings generate lift, and set to 1.2 throughout, based on aeronautical arguments as in the majority of avian flight studies [53]. From Equation (10) it is possible to find the unique value of *V* that minimises the sum of *P*_par_ and *P*_ind_. The final component of power output, profile power *P*_pro_, is estimated as 1.2 times this minimal sum of *P*_par_ and *P*_ind_ as given by Equations (9) and (10). From the above, the total mechanical power expended in flight is simply the sum *P*_par_ + *P*_ind_ + *P*_pro_. Although estimation of profile power in this way is common, it is too simple to be fully realistic because it does not allow *P*_pro_ to vary with flight speed. However, when dealing with extinct taxa for which wing kinematics are not known, it is the only available option.

In the above calculations, we assumed that the costs of ploughing the tip of the mandible through the water and of flight were not separable, and should not be simply summed or ignored as they have been in previous studies where mandible drag was considered negligible [19,31,32]. We therefore included mandible drag in the flight power model as additional parasite drag as recommended by Ward et al. [50] for drag due to respirometry masks in flight studies to take account of its effects on performance in addition to the direct drag costs.

We estimated the total power consumption of flight by using the aerodynamic equations to calculate the mechanical component of power output during flight (*P*_mech_) and obtain an estimate of *P*_met_ from this by:

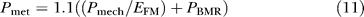
where flight muscle efficiency (*E*_FM_) is defined as *P*_mech_/metabolic power consumed by the flight muscles, and *P*_BMR_ is basal metabolism ([47,49] and see [50] for a discussion of the errors associated with this estimate]. The constant value of 1.1 results from the assumption that the extra costs of respiration and circulation during flight each contribute 5% to *P*_met_ [47]. A recent study examining *E*_FM_ in starlings *(Sturnus vulgaris)* suggested values of around 0.18 for birds in the size range of starlings (approximately 0.1 kg [50]). However, in the absence of other data, and given the known increase in efficiency with size, we used the commonly used value of 0.23 (e.g., [47]). We used an allometric equation for non-passerines (*P*_BMR_ = 3.8 *M* ^0.72^, [54]) to estimate *P*_BMR_ from mass.

As a further step, a method for calculating the cost of flying close to the ground (wing-in-ground effect) is presented by Rayner [30]. From published photographs of foraging skimmers (e.g., [17]) we estimated that when held parallel to the water's surface, the skimmer's wing is some 10 cm above the water. This corresponds to the height estimated from considering the geometry of the bird in flight, i.e., (*L* − *d*) cos(45°), where *L* is mandible length and *d* is immersion depth. From this we calculated a relative height as β = *h*/*b*, where *h* is the height of the wing from the water surface and *b* the wing semispan. Next, the aspect ratio of the wings *r*_W_ is calculated as 4*b* ^2^/*S*, and from this, the minimum drag-to-weight ratio,

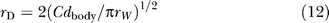



Two further coefficients denote the reduction in induced drag due to the ground effect (σ), and a circulation factor due to ground effect (τ). These coefficients are graphed as functions of β in Figure 2 of Rayner [30].

From Equation (11), we identify *V*_mp_, the velocity that minimises energetic costs (away from the ground effect). We can then calculate the costs of flying at any speed *V* correcting for the effects of flying near the ground *P*_ge_ according to

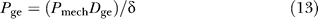
where *D*_ge_ is the total drag of the animal corrected for ground effect, and δ is the relative drag,

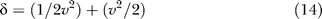
where *v* is the scaled velocity *V*/*V*_mp_. To calculate *D*_ge_, we first calculate γ, the relative circulation [30]:

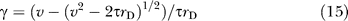



Then

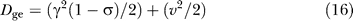
Flap-gliding or intermittently powered flight is an energy-saving behaviour and is frequently seen in *Rynchops* [19,31]. Flap-gliding is characterised by the use of powered flight to increase height before gliding using open wings. During the gliding phase, the bird supports its weight with its wings but does no mechanical work; it loses height as potential energy is converted into work against drag. The resulting undulating flight path was approximated by the following method, based on that of Norberg [49]. The assumption was made that the minimum power velocity for powered flight (*V*_mp_) was equal to both the glide (*V*_glide_) and climb (*V*_climb_) velocities. From this it follows that the vertical sinking speed of the animal at a glide angle φ is

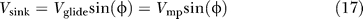



The backward drag component *D* in this situation is approximated by

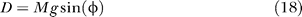



The proportion of time spent climbing from the water surface to elevation *h* is given by

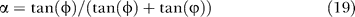
where ϕ is the slide angle. The mechanical power required to climb using powered flight is given by

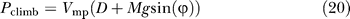



To produce an estimate of flight costs during flap-gliding, climb angle ϕ was set at 45 ° and φ was adjusted to minimise the cost of flight at *V*_climb_ (i.e., minimise the product α *P*_climb_), with the limitation that φ ranged from 1 ° to 30 ° (corresponding to reasonable glide angles for extant volant animals and human gliders [33]). The total average power required for undulating flight is then simply given by the sum of metabolic power required to climb, power required for forward flight with the mandible in the water, and metabolic costs of basal metabolic rate during gliding

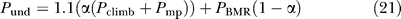



It should also be noted that this method does not account for changing drag costs associated with variable mandible submersion, drag of the mandible while gliding, or changes in flight costs due to impacts of elevation on wing-in-ground effect. It does, however, give a rough estimate of the flight costs during flap-gliding for comparison with powered level flight.

### Sensitivity analysis.

To evaluate the sensitivity of our aerodynamic model, we varied the values of *k* and *Cd*_body_ to simulate high- and low-power cases for *Rynchops, Tupuxuara,* and *Thalassodromeus* (holotype) following [55] and [56]. For the low-power case, *k* was decreased from 1.2 to 1.0 and *Cd*_body_ was reduced by 60% to 0.16. In the high-power case, *k* was increased to 1.4 while *Cd*_body_ was increased by 60% to 0.40. These values allowed us to span the range of current estimates for these two parameters and so bracket the power outputs for a given flight speed (see shaded regions in Figure 3).

## Abbreviations

*Fr*: Froude number
OLS: ordinary least-squares regression
*Re*: Reynolds number

## References

[pbio-0050204-b001] Chatterjee S, Templin RJ (2004). Posture, locomotion, and paleoecology of pterosaurs.

[pbio-0050204-b002] Kellner AWA, Campos DA (2002). The function of the cranial crest and jaw of a unique pterosaur from the Early Cretaceous of Brazil. Science.

[pbio-0050204-b003] Ruxton GD, Houston DC (2003). Could Tyrannosaurus rex have been a scavenger rather than a predator? An energetics approach. Proc R Soc Lond B.

[pbio-0050204-b004] Mateer NJ (1975). A study of *Pteranodon*. Bull Geol Inst University Uppsala.

[pbio-0050204-b005] Price S (2006). Extrapolating from extant to extinct: Reconstruction of early Neocete life-history strategy and ecology. J Vert Paleon.

[pbio-0050204-b006] Prieto IR (1998). Morfología funcional y hábitos alimentarios de Quetzalcoatlus (Pterosauria). Coloquios de Paleontologia.

[pbio-0050204-b007] Wellnhofer P (1980). Flugaurierreste aus der Gosau-Kreide von Muthnannsdorf (Niererösterreich) - ein Beitrag zur Kiefermechanik der Pterosaurier. Mitt Bayer Staatsslg Paläont Hist Geol.

[pbio-0050204-b008] Gillooly JF, Allen AP, Charnov EL (2006). Dinosaur fossils predict body temperatures. PLoS Biol.

[pbio-0050204-b009] Massare JA (1987). Tooth morphology and prey preference of Mesozoic marine reptiles. J Vert Paleon.

[pbio-0050204-b010] Rayfield EJ (2004). Cranial mechanics and feeding in Tyrannosaurus rex. Proc R Soc Lond B.

[pbio-0050204-b011] Taylor MA (1992). Functional anatomy of the head of the large aquatic predator Rhomaleosaurus zetlandicus (Plesiosauria, Reptilia) from the Toarcian (Lower Jurassic) of Yorkshire, England. Phil Trans Biol Sci Lond B.

[pbio-0050204-b012] Abel O (1912). Grundzüge der Paläobiologie der Wirbeltiere.

[pbio-0050204-b013] Kellner AWA, Langston W (1996). Cranial remains of *Quetzalcoatlus* (Pterosauria, Azhdarchidae) from Late Cretaceous sediments of Big Bend National Park, Texas. J Vert Paleont.

[pbio-0050204-b014] Martill DM (1997). From hypothesis to fact in a flight of fancy: The responsibility of the popular scientific media. Geol Today.

[pbio-0050204-b015] Nesov LA (1984). Pterosaurs and birds of the Late Cretaceous of Central Asia. Palaontologische Zeitschrift.

[pbio-0050204-b016] Wellnhofer P (1991). Illustrated encyclopedia of Pterosaurs.

[pbio-0050204-b017] Zusi RL, del Hoyo J, Elliott A, Sargatal J (1996). Handbook of the birds of the world, vol. 3.

[pbio-0050204-b018] Tompkins IR (1963). Skimmer-like behavior in the Royal and Caspian terns. Auk.

[pbio-0050204-b019] Withers PC, Timko PL (1977). The significance of ground effect to the aerodynamic cost of flight and energetics of the black skimmer (Rynchops nigra). J Exp Biol.

[pbio-0050204-b020] Black BB, Harris LD (1983). Feeding habitat of black skimmers wintering on the Florida Gulf coast. Wilson Bull.

[pbio-0050204-b021] Erwin RM (1977). Foraging and breeding adaptations to different food regimes in three seabirds. Ecology.

[pbio-0050204-b022] Potter JK (1932). Fishing ability of the black skimmer (*Rynchops nigra nigra*). Auk.

[pbio-0050204-b023] Tompkins IR (1951). Method of feeding of the black skimmer, Rynchops nigra. Auk.

[pbio-0050204-b024] Zusi RL (1962). Structural adaptations of the head and neck in the Black Skimmer, Rynchops nigra Linnaeus. Publications of the Nuttal Ornithological Club.

[pbio-0050204-b025] Arthur SC (1921). The feeding habits of the black skimmer. Auk.

[pbio-0050204-b026] Bennett SC (2001). The osteology and functional morphology of the Late Cretaceous pterosaur *Pteranodon*. (parts 1 and 2). Paleontographica Abt A.

[pbio-0050204-b027] Ősi A, Weishampel DB, Jianu CM (2005). First evidence of azhdarchid pterosaurs from the Late Cretaceous of Hungary. Acta Palaeo Polonica.

[pbio-0050204-b028] Veldmeijer AJ, Signore M, Meijer HJM (2005). Description of two pterosaur (Pterodactyloidea) mandibles from the lower Cretaceous Santana Formation, Brazil. Deinsea.

[pbio-0050204-b029] Marden JH (1994). From damselflies to pterosaurs: How burst and sustainable flight performance scale with size. Am J Physiol.

[pbio-0050204-b030] Rayner JMV (1991). On the aerodynamics of animal flight in ground effect. Phil Trans R Soc Lon B.

[pbio-0050204-b031] Blake RW (1984). A model of foraging efficiency and daily energy budget in the Black Skimmer (Rynchops nigra). Can J Zool.

[pbio-0050204-b032] de la Cueva H, Blake RW (1993). Mechanics and energetics of ground effect in flapping flight. Contemp Math.

[pbio-0050204-b033] Vogel S (1994). Life in moving fluids: The physical biology of flow.

[pbio-0050204-b034] Hazelhurst GA, Rayner JMV (1992). Flight characteristics of Triassic and Jurassic Pterosauria: An appraisal based on wing shape. Paleobiology.

[pbio-0050204-b035] Berner RA, Canfield DE (1989). A new model for atmospheric oxygen over phanerozoic time. Am J Sci.

[pbio-0050204-b036] Graham JB, Dudley R, Aguilar NM, Gans C (1995). Implications of the late Palaeozoic oxygen pulse for physiology and evolution. Nature.

[pbio-0050204-b037] Bock WJ (1960). Secondary articulation of the avian mandible. Auk.

[pbio-0050204-b038] Wellnhofer P (1975). Die Rhamphorhynchoidea (Pterosauria) der Oberjura-Plattenkalke Süddeutschlands. Teil I. Palaeontographica A.

[pbio-0050204-b039] Howse SCB (1986). On the cervical vertebrae of the Pterodactyloidea (Reptilia, Archosauria). Zool J Linnea Soc.

[pbio-0050204-b040] Frey E, Martill DM (1996). A reappraisal of *Arambourgiania* (Pterosauria, Pterodactyloidea): One of the world's largest flying animals. Neues Jahrbuch für Geologie und Paläontologie, Abhandlungen.

[pbio-0050204-b041] Lawson DA (1975). Pterosaur from the Latest Cretaceous of West Texas: Discovery of the largest flying creature. Science.

[pbio-0050204-b042] Hoerner SF (1965). Fluid-dynamic drag.

[pbio-0050204-b043] Fish FE, Blood BR, Clark BD (1991). Hydrodynamics of the feet of fish-catching bats: Influence of the water surface on drag and morphological design. J Exp Zool.

[pbio-0050204-b044] Sheldahl RE, Klimas PC (1981). Aerodynamic characteristics of seven airfoil sections through 180-degree angle of attack for use in aerodynamic analysis of vertical axis wind turbines. Report SAND80–2114.

[pbio-0050204-b045] Heptonstall WB (1971). An analysis of the flight of the Cretaceous pterodactyl Pteranodon ingens. Scott J Geol.

[pbio-0050204-b046] Paul GS (2002). Dinosaurs of the air: The evolution and loss of flight in dinosaurs and birds.

[pbio-0050204-b047] Pennycuick CJ (1989). Bird flight performance.

[pbio-0050204-b048] Hedenström A (2002). Aerodynamics, evolution and ecology of avian flight. Trends Ecol Evol.

[pbio-0050204-b049] Norberg UM (1990). Vertebrate flight.

[pbio-0050204-b050] Ward S, Möller U, Rayner JMV, Jackson DM, Bilo D (2001). Metabolic power, mechanical power and efficiency during wind tunnel flight by the European starling Sturnus vulgaris. J Exp Biol.

[pbio-0050204-b051] Hedenström A, Rosén M (2003). Body frontal area in passerine birds. J Avian Biol.

[pbio-0050204-b052] Nudds RL, Rayner JMV (2005). Scaling of body frontal area and body width in birds. J Morph.

[pbio-0050204-b053] Pennycuick CJ, Klaassen M, Kvist A, Å L (1996). Wingbeat frequency and the body drag anomaly: wind-tunnel observations on a thrush nightingale (Luscinia luscinia) and a teal (Anas crecca). J Exp Biol.

[pbio-0050204-b054] Schmidt-Nielson K (1984). Scaling: Why is animal size so important?.

[pbio-0050204-b055] Tobalske BW, Hedrick TL, Dial KP, Biewener AA (2003). Comparative power curves in bird flight. Nature.

[pbio-0050204-b056] Spedding GR, Pennycuick CJ (2001). Uncertainty calculations for theoretical flight power curves. J Theor Biol.

